# Lateral supracerebellar infratentorial approach for pontomesencephalic cavernous malformations

**DOI:** 10.3171/2019.7.FocusVid.191227

**Published:** 2019-07-01

**Authors:** Karl R. Abi-Aad, Devi P. Patra, Matthew E. Welz, Evelyn Turcotte, Bernard R. Bendok

**Affiliations:** Departments of 1 Neurological Surgery,; 2 Otolaryngology, and; 3 Radiology, Mayo Clinic;; 4 Precision Neuro-therapeutics Innovation Lab, Mayo Clinic; and; 5 Neurosurgery Simulation and Innovation Lab, Mayo Clinic, Phoenix, Arizona

**Keywords:** lateral supracerebellar infratentorial, brainstem cavernoma, surgical anatomy, video

## Abstract

Cavernomas at the posterolateral pontomesencephalic surface can be approached from a lateral infratentorial supracerebellar corridor. In this surgical video, we demonstrate two cases of brainstem cavernomas resected through a lateral supracerebellar infratentorial approach. A supine position with lateral turn of the head was used along with significant reverse Trendelenburg to allow the cerebellum to fall away with gravity from the tentorium. After exposure of the posterior surface of the brainstem between the tentorium and the superior cerebellar surface with aid of neuronavigation, the cavernomas were safely resected.

The video can be found here: https://youtu.be/fUDdaprg26Y.

## Transcript

My name is Bernard Bendok and I’ll be showing two cases that illustrate the lateral supracerebellar infratentorial approach to pontomesencephalic cavernous malformations.

The first case involves a 59-year-old male with slurred speech and right-sided weakness, who had this growing left pontomesencephalic junction cavernoma. The lesion came to the surface.

The two approaches that can be considered for this lesion are a lateral supracerebellar infratentorial approach or a subtemporal approach. Both have pros and cons. The advantage of the lateral supracerebellar approach is that it brings you in direct view of the lateral mesencephalic sulcus, which is a safe entry zone to the brainstem.

This approach, also in the case that we’re presenting, brought us closest to the area of this cavernoma that presented to the surface of the brainstem. One has to pay special attention to the fourth cranial nerve, which is typically at the inferior edge of the exposure. A subtemporal approach is an alternative, but it would come in more lateral than ideal for a lesion that projects and comes to the surface in this direction.

The advantages of the subtemporal approach are that it brings you in direct contact with the anterolateral midbrain, but retraction in this location can risk injury to the vein of Labbé and to temporal lobe itself. The lateral supracerebellar infratentorial approach, on the other hand, when combined with significant reverse Trendelenburg, results in minimal cerebellar retraction as the cerebellum descends with gravity, especially after CSF release. There are situations where bridging veins could limit the exposure from this trajectory.

The lateral mesencephalic sulcus has been defined in the literature as a safe entry zone to the brainstem.

When looking at cross section, the substantia nigra is anterior and lateral, and the ocular motor nerve is anterior and medial, and the medial lemniscus is medial to this sulcus. Clearly, one has to be careful during the dissection, using image navigation. In my view, the closer the lesion comes to the surface, the better in terms of safety.

This is the surgical approach, as illustrated in this video. We typically use a horseshoe incision, although a linear incision can also be used. The transverse sinus is exposed so that the dural leaflet can retract the transverse sinus superiorly, thus widening the exposure.

We typically use significant reverse Trendelenburg to allow cerebellar descent with gravity.

In this video, the arachnoid planes are being opened exposing the midbrain. Cavernoma appears to be exophytic. And here with careful bipolar and suction, the cavernoma could be removed piecemeal, being careful to avoid unnecessary traction on brainstem tissue. It is very important to preserve developmental venous anomalies, and to avoid injury to venous structures.

This postoperative scan shows complete resection of the cavernoma. The patient was neurologically at baseline after the procedure.

The second case involves a 54-year-old female with a history growing brainstem cavernoma at the right pontomesencephalic junction.

We approach this lesion through a lateral supracerebellar infratentorial approach. Two small bridging veins were divided and using image guidance, we were able to locate the area of the brainstem where the cavernoma came closest to the surface. Bipolar was used to shrink the cavernoma and it was removed piecemeal, being careful to avoid any manipulation of the normal brainstem tissue.

Her postoperative MRI showed complete resection and she was neurologically intact after the procedure.

The lateral supracerebellar infratentorial approach when combined with significant reverse Trendelenburg with the patient in a supine head-turned position provides a natural corridor below the tentorium to the posterolateral aspect of the pontomesencephalic junction.

Revere Trendelenburg positioning helps in widening of the corridor due to gravity.

CSF drainage from the CP angle cistern and cisterna magna further enhances cerebellar relaxation.

Attention must be paid to venous anatomy that may hinder the approach or contribute to postoperative complications.

### Time points

0:42 Approaches to the lateral pontomesencephalon
1:00 Illustration of the lateral supracerebellar infratentorial surgical corridor
1:17 Illustration of the subtemporal surgical corridor
1:28 Advantages and disadvantages of the lateral supracerebellar infratentorial and subtemporal approaches
1:58 Cross section of the midbrain that delineates the borders of the lateral mesencephalic sulcus
2:20 Fly through showing the lateral supracerebellar infratentorial approach
2:36 Patient positioning
2:46 Surgical video of cavernoma resection
4:20 Pearls that address the lateral supracerebellar infratentorial approach
